# Some Cardiac Problems*Read at the annual meeting of the Lake Keuka Medical and Surgical Association held at Keuka, N. Y., July 17 and 18, 1913.

**Published:** 1913-12

**Authors:** John M. Swan

**Affiliations:** Rochester, New York


### BUFFALO MEDICAL JOURNAL

VOLUME 69 DECEMBER, 1913 No. 5

### ORIGINAL ARTICLES

*The right is reserved to decline papers not dealing with practical medical and surgical subjects and such as might offend or fail to interest readers. Contributors are solely responsible for opinions, methods of expression and revision of proof.*

### Some Cardiac Problems*

By JOHN M. SWAN, M D., OF ROCHESTER, NEW YORK.

AMONG the cases belonging to the field of the internist none present more troublesome or more important problems for solution than the cases of disease of the heart. The troublesome element in these problems is emphasized by the frequent reports in the newspapers of cases of sudden death from heart disease, so that a person who has been told that he has heart disease is likely to get the idea that his disease, also, must end suddenly. This idea has been possibly more strongly impressed on the lay mind within the last year by the production of a play, "The Return of Peter Grimm," in which a cardiac death is reproduced before the eyes of the audience.

I. VALVULAR LESIONS.

One of the first problems then that must be answered in a heart case is whether, because an individual carries a valvular lesion, he is bound to drop dead. The existence of a valvular lesion will not necessarily prove more than an annoyance, demanding a little extra care in the ordering of the patient's life; but not demanding medication or rigid abstinence from the ordinary activities so long as compensation is maintained.

Case 1.—Female, aged 24 years, who complained of being afraid she was going to die because she had been told she had heart disease. She was in the habit of reading all the reports in the newspapers about persons who died suddenly from heart failure and about disasters of various kinds. Then she would lie awake at night and worry about her condition. She complained of precordial distress and noticed that her heart occasionally dropped a beat. But she presented none of the symptoms of decompensation such as vertigo, dyspnea, palpitation, edema and cough. This patient had a well compensated mitral regurgitation. The murmur was loud, and distinctly transmitted in the normal way; her liver was not enlarged; her systolic blood pressure was 138 mm; her pulse pressure was 60 mm; her pulse 80. She was subject to frequent attacks of tonsilitis and to hysterical attacks. One month after she was first seen she had an attack of tonsilitis; she had a second attack one month after the first attack, during which her temperature went to 102° and her pulse to 130. During this attack she developed a systolic thrill at the apex and an aortic diastolic murmur; both of which disappeared with the cessation of the attack. Ten months after she was first seen she fainted one morning after she had eaten an indigestible supper after visiting the theater; she had also been constipated at that time for several days. There was no change in the heart condition, however, and her pulse was 78 per minute when seen. I have never been able to detect an irregular pulse in this patient.

The problems in this case are: (1) Whether the mitral lesion justified a prognosis which so frightened the patient that she became hysterical. (2) Whether the precordial distress and the sensation of irregularity were due to the heart disease or to functional nervous disturbance. (3) Whether such a patient should have her tonsils removed. I cannot deprecate too strongly the tendency which I have observed in some quarters to give a serious prognosis upon the discovery of a cardiac murmur. I attribute the very unenviable condition of this patient when I first saw her to the very pessimistic outlook which she had been given by several men who had examined her. A well compensated mitral lesion may persist for years without decompensation if the patient lives an ordinarily careful life. On the other hand, it is just as reprehensible to let a patient with a valvular defect think he can indulge in athletic exercises and other extreme activities. In this case I am inclined to attribute the precordial distress and the sensation of irregularity to nervous influences. I am of the opinion that in a case of this kind tonsillectomy would better be done to avoid the danger of the toxic influence of repeated infections upon the hypertrophied heart muscle.

Case 2.—In contrast to this case is that of a man, aged 50 years, who had had several attacks of palpitation, dyspnea, precordial pain and vertigo. He had mitral regurgitation, which was well compensated. His cardiac dullness measured 18 cm. in oblique diameter; his systolic blood pressure was 125 mm.; his pulse pressure was 57 mm.; his pulse 68, regular, full and strong. Three months after his first examination he had an attack of vertigo, deafness in the left ear, weakness, nausea and chilliness. On examination the oblique diameter of cardiac dullness was found to be 19.4 cm. There was a presystolic thrill; a presystolic rumble, followed by a soft systolic murmur which was heard all over the precordium and in the axilla. There was a soft blowing systolic murmur at the base of the heart, which was not transmitted into the vessels of the neck; and a soft systolic murmur at the ensiform cartilage. The edge of the liver was palpable. The systolic blood pressure was 118 mm.; the pulse pressure 58 mm.; the pulse 64 in the recumbent posture. I attributed this attack to a not very marked acute dilation of the heart. Rest in bed resulted in a return of the oblique diameter of the cardiac dullness to 17.5 cm., and the systolic blood pressure to 124 mm. During his period of confinement to bed, while sitting up one morning to have his bed made, he became weak and complained of vertigo. That night he felt chilly and had a febrile attack with the symptoms of gastro-intestinal disturbance. His urine at this time contained pus, epithelium, bacteria, uric acid, calcium oxalate crystals and a few hyaline casts without albumin. A rectal examination showed an enlarged and tender prostate gland.

The problems in this case are: (1) Was the attack diagnosticated acute dilation an acute dilation in fact. (2) What influence had the prostatitis on his cardiac condition. The increase in oblique diameter of cardiac dullness from 18 cm. to 19.4 cm., may be within the limit of error in outlining the heart by percussion; but the appearance of the presystolic thrill, of a presystolic element to a murmur which had been purely late systolic, and a systolic murmur at the ensiform cartilage; a fall of blood pressure, and an increase in the size of the liver so that its edge became palpable, may be taken as sufficient evidence of a marked disturbance in the cardiac physiology and confirm the diagnosis. The prostatitis undoubtedly accounted for the septic attack experienced while the patient was in bed and illustrates the advisibility of studying the patient from all points of view; not being content with the establishment of one lesion and overlooking others that may be present. Septic absorption from a chronically inflamed prostate is quite sufficient to disturb the function of a normal heart and can do more damage to one the seat of mitral disease.

### II. Irregularities.

While the problem of the proper advice to give to a patient who carries a well compensated valvular lesion is often difficult of solution; indeed, it is not always easy to be sure that a valvular lesion is compensated; the problem of the significance of a cardiac irregularity is even more serious and troublesome. There is, perhaps, no more alarming subjective symptom in medicine than the sensation of stopping of the heart, dropping of beats, or any other disturbance of its rhythm. But every cardiac irregularity is not indicative of myocardial change, as some appear to think. Many irregularities are the result of extracardiac influence, often of a toxic nature, and quite amenable to treatment.

Case 3.—Male, aged 65 years, contractor, complained of cardiac irregularity. He had been conscious of the dropping of beats for two years, and he described the sensation as a "fluttering." He had had some attacks of vertigo; some dyspnea on exertion, without palpitation; some retrosternal pain on walking too fast; an occasional attack of "sour stomach," which was relieved by vomiting; and some indefinite abdominal pain. He also complained of insomnia. The patient was a very large man, who weighed 239 pounds. He was a large eater of carbohydrates, and claimed to be a moderate user of alcohol on social occasions. For many years he had been addicted to the excessive use of tobacco; he smoked eight to ten cigars a day and chewed a pound of tobacco a month. Two years before I saw him he stopped his tobacco and had used none since. The physical examination showed a slight pulmonary emphysema and some edema of the ankles in addition to the circulatory condition, which was as follows: "Heart: P. M. I., not obtainable. Dullness, 3d interspace, 6th rib, 5 cm. to the right of the midsternal line, 17.5 cm. to the left of the midsternal line. Oblique diameter of cardiac dullness 25.5 cm. There is a soft systolic murmur at the apex, not transmitted and a loud systolic murmur at the base of the heart not transmitted; but heard louder at the right than at the left edge of the sternum. The muscular quality of the systolic sound is deficient. Blood pressure: Systolic, 144; diastolic, 92; pulse pressure, 52. Pulse, 68, dropped five beats in one-half minute, fair strength and volume, arteries thickened." His urine had a specific gravity of 1.017, and contained neither albumin nor glucose, but there were a few hyaline and pale granular casts in the sediment.

The problem in this case was to determine whether the irregularity was due to a myocarditis or to some toxic influence acting on the heart. The man was very large, and his heart was larger than it should be even for a man of his size. The only important signs of decompensation were the apical murmur and the edema of the ankles. I decided to proceed on the theory that the irregularity was toxic, and so ordered eliminative treatment. He had an electric light bath for ten minutes, followed by a blanket pack for one-half hour, a spray, and an hour's rest three times a week. His carbohydrate intake was reduced. After one month of this routine, although he felt well generally, there was no improvement in the irregularity. The edema had entirely disappeared; but he still complained of dyspnea on exertion and of rheumatic pains. His pulse dropped five beats in one-half minute. His blood pressure was: Systolic, 150; diastolic, 84; pulse pressure, 70; pulse, 75. He was then put on ten minims of tincture of digitalis and one minim of spts. glycerylis nitratis twice a day. At the end of two months of this medication the irregularity was still present. The oblique diameter of cardiac dullness was 24.5 cm.; the murmurs were still present, and the muscular quality of the systolic sound was still markedly deficient. His pulse was 60, and dropped every third beat. He was then put on sodium iodide, five grains after each meal. In two weeks the irregularity disappeared and has not been noted since, more than one year. The sodium iodide was continued for eight months. At the last examination the oblique diameter of his heart was 21 cm. The systolic, apical murmur had disappeared. The last observation of the blood pressure gave systolic, 145; diastolic, 102; pulse pressure, 42. The pulse was 82 and regular. His urine was still of low specific gravity, but at the last examination contained neither albumin nor casts.

Irregularities due to muscle disease are alarming and require great care and careful handling on the part of the physician. In order to have all obtainable evidence at hand, the irregularity ought to be studied by means of the polygraph or by the electrocardiograph, if one is obtainable. An irregularity that shows a prolonged a-c interval, I believe, ought never to be treated with digitalis.

Case 4.—Male, aged 42 years, had known that he had an irregular heart for sixteen or seventeen years. When I first saw him he was in a very nervous condition because he had received so many unfavorable prognoses that he had worried a great deal for fear that he might die suddenly. The patient led a sedentary life without excesses. He was of constipated habit. He had had two attacks, characterized by pain in his legs and unsteadiness of gait; but not accompanied by numbness or loss of power. He complained of feeling well in the morning, but easily getting tired during the day; his head felt heavy; he complained of palpitation after eating a heavy meal; of gas in his intestines and of cold feet. The patient was slightly overweight. His chest was assymmetrical. He had a marked scoliosis in the lower thoracic and lumbar regions, the result of falling down stairs when he was a boy. His heart was of normal size; there were no murmurs, and the muscular quality of the systolic sound was good. The blood pressure study showed systolic, 160; diastolic, 103; pulse pressure, 57. Pulse, 72; dropped five beats in one-half minute, small, fair strength, arteries not palpable.

I felt, on account of the normal size of this patient's heart, the absence of murmurs, and the good muscular element of the systolic sound, that this irregularity was not due to myocardial change. His urine showed a specific gravity of 1.028; no albumin, no glucose, but calcium oxalate and uric acid crystals and a few hyaline casts. This evidence of disordered metabolism and his constipation led me to look on this as a toxic case, and I put him on an electric light bath, ten minutes, followed by a spray at 95°, general massage, particularly to the back, followed by an hour's rest three times a week. At the end of a month the patient was less nervous, he noticed the irregularities much less, his blood pressure was: systolic, 142; diastolic, 105; pulse pressure, 37; pulse, 76; but the irregularity was still present; there was a drop of two beats every half minute. This irregularity has continued. I still look on the case as one that need give no uneasiness at present, at least from the cardiac side. I am inclined to believe that the deformity of the thorax, occasioned by the scoliosis, has some causative influence on the irregularity; sodium iodide had no effect on it.

Case 5.—A female patient, aged 59 years, complained of frequent attacks of palpitation of the heart and irregularity, of about one year's duration. The attacks began after being tired and rundown for several weeks, but the exciting cause was climbing a short hill. She had cataracts beginning in both eyes, dry mouth, dyspnea, and she was quite nervous. Her expression was anxious, no exophthalamus, no eye signs; the thyroid body was palpable. The heart was not hypertrophied; there were no murmurs, but the heart's action was very irregular. Blood pressure, systolic, 152; diastolic, 40; pulse pressure, 112. Pulse, 116; irregular. No gross arterial thickening. The stomach was slightly dilated. The attacks of palpitation and irregularity usually began in the morning or during the night. There was no angina, no edema. There was a fine tremor in both hands. Her blood showed a slight chloroanemia with 31.2 per cent. lymphocytes and 14.4 per cent. large mononuclears. Her urine contained a trace of albumin on one occasion and a trace of glucose on another occasion. There was a large number of uric acid crystals in the sediment but no casts. I attributed this palpitation and irregularity to toxic influence, either gastrointestinal or thyroid. Dr. M. B. Palmer made a radiographic examination after a bismuth meal, and reported the gastrointestinal tract normal. After excluding a gastrointestinal influence the patient was put to bed, and an ice bag was applied to the precordium for three weeks, when the right superior thyroid artery was ligated and cut by Dr. M. B. Tinker of Ithaca. After this operation the attacks of irregularity ceased, but the pulse continued rapid. That is, out of fifty-one observations twenty-six were above ninety, although at the time the patient left the hospital, three weeks after the operation, the rate was between seventy-two and ninety the greater part of the time. On June 30th the left superior thyroid artery was ligated and cut. After this operation the pulse was irregular, the patient complained a good deal of dyspnea and the pulse was rapid, above ninety sixteen times out of twenty-six observations. The patient died suddenly early in the morning of July 9th. The question of the wisdom of advising surgical procedure in this case comes up. I felt that the cardiac symptoms were dependent upon thyroid influence. The absence of gross heart changes and of palpable peripheral vessels makes me believe that the cardiac symptoms were due to extracardiac influence and not to myocardial change. The high blood pressure might very well be due to thyroid influence. The sudden, unexplainable death might be due to auricular filtration.

### III. Retrosternal Pain.

The last problem that I wish to touch upon is that of the opinion to be given in cases of precordial and retrosternal pain. Shall we, as some writers would seem to advocate, make a diagnosis of angina pectoris in every case of pain in the precordium; or shall we reserve that diagnosis for typical attacks only; those characterized by sudden, sharp pain with classical radiation and fear of impending death?

It seems to me that so far as the patient is concerned he would better be reassured whenever possible. I would prefer to tell a patient he has not angina pectoris when I thought he might have than to tell him he had it if I had the least doubt in my mind. On the other hand, some member of the family in such a case ought to be warned of the possibilities.

Case 6.—Male, aged 63 years, complained of retrosternal pain of two years duration. The attacks of pain came on whenever he walked or made any exertion; the pain was sharp, radiated into both arms, and was accompanied by flushing of the face. There was no sense of impending death. The cold weather made his attacks more severe and more frequent. He sometimes had three or four attacks of pain in one day; but always one attack a day. Sometimes walking 1000 feet would bring on an attack; some of the attacks came on when he was undressing. He complained of a morning cough, with a little expectoration, which had been bloodstained, and dyspnea and palpitation of the heart on exertion; but none of the other signs of decompensation. The physical examination showed a rather thin man, with a sallow complexion and a slight amount of suborbital puffiness. There was moderate pulmonary emphysema. The examination of the heart is recorded in my notes as follows: "P. M. I. not obtainable. Dullness, 3d rib, 5th interspace, 2 cm. to the right of the midsternal line, 10.5 cm. to the left of the midsternal line. Oblique diameter of cardiac dullness 16 cm. There are no murmurs. The diastolic sounds are equal in intensity. The muscular quality of the systolic sound is decidedly deficient."

There were no alterations demonstrable in the abdominal organs. The blood pressure determinations gave systolic, 129; diastolic, 74; pulse pressure, 55. The pulse was 82, regular, good strength and volume. On assuming the upright from the recumbent posture the pulse decreased to 76. His urine was rather high in specific gravity; contained no albumin and no glucose, but the sediment showed a few hyaline casts and uric acid crystals. There was a marked excess of indican.

This patient had consulted many physicians; he was worried and anxious on account of the diagnosis of angina pectoris that had been made by some of the physicians consulted. On account of the absence of cardiac enlargement; on account of the absence of symptoms of serious decompensation, to be sure he had dyspnea and palpitation on exertion, and on account of the low blood pressure, I told him positively that he did not have angina pectoris. I started him on a course of carborated brine baths, which did him no good, and a short time after I saw him last he died of cardiac failure after a typical attack of angina. In this case I failed to give proper weight to the fact that the attacks of pain were brought on by exertion, to the low blood pressure, and to the marked deficiency of the muscular quality of the systolic sound of the heart.

457 Park Avenue.

---

*Read at the annual meeting of the Lake Keuka Medical and Surgical Association held at Keuka, N, Y., July 17 and 18, 1913.

